# Primary sigmoid vaginoplasty in gender affirmation surgery: technique and outcomes

**DOI:** 10.1093/sexmed/qfag007

**Published:** 2026-04-16

**Authors:** N Arjun, Y S Nataraj, Dinesh Anne, Bhavya Shetty, H K Nagaraj

**Affiliations:** Department of Urology, Sapthagiri Medical College, Bangalore, Karnataka, 560090, India; Department of Surgical Oncology, Sapthagiri Medical College, Bangalore, Karnataka, 560090, India; Department of Urology, Sapthagiri Medical College, Bangalore, Karnataka, 560090, India; Department of Urology, Sapthagiri Medical College, Bangalore, Karnataka, 560090, India; The Bangalore Kidney Stones Hospital, Bangalore, Karnataka, 560090, India

**Keywords:** gender affirmation surgery, transgender surgery, sigmoid vaginoplasty, vaginal reconstruction, gender reassignment surgery

## Abstract

**Background:**

Vaginal canal reconstruction is critical for transgender and gender-diverse individuals assigned male at birth, seeking gender affirmation, but conventional techniques often have limitations in depth and tissue quality.

**Aim:**

The aim was to evaluate outcomes of primary sigmoid vaginoplasty as a gender-affirming surgical option for transgender women at a tertiary center.

**Methods:**

A prospective review included transgender women operated for primary sigmoid vaginoplasty from 2023 to 2025, with data collected on demographics, perioperative details, complications, and postoperative neovaginal depth; assessment relied on physical examination, preoperative screening, standardized surgical technique, and postoperative follow-up with dilation routines.

**Outcomes:**

Primary outcomes were neovaginal depth, maintenance of sexual function, and postoperative complications.

**Results:**

Thirty patients achieved a mean neovaginal depth of 16 ± 2.0 cm intraoperatively; all maintained sexually functional tissue. There were 2 major complications—1 bowel injury and 1 anastomotic leak—requiring reoperation, with no mortality. Introital stenosis occurred in 10% and was successfully treated with dilation. Average hospital stay was 8 ± 2.1 days, and long-term follow-up confirmed satisfaction with sexual function and depth, without significant odor or excessive secretions.

**Clinical Implications:**

Sigmoid vaginoplasty offers a reliable approach for achieving functional vaginal depth and patient satisfaction, particularly in individuals with limited penile tissue. Bowel Complications can set in, requiring adequate evaluation, motivation and understanding of the procedure.

**Strengths and Limitations:**

Strengths include the prospective design and standardized surgical technique, while limitations are the small sample size, single-center scope, and limited follow-up duration for some patients due to geographic constraints.

**Conclusion:**

Primary sigmoid vaginoplasty is an effective and dependable surgical option for transgender women, yielding satisfactory depth, appearance, and sexual function but can come with associated bowel complications.

## Introduction

Gender-affirming surgery has become a recognized component of the transition process for transgender individuals, even though not all Transgender Individuals opt for surgery as part of their transition. [1]. These surgeries enhance quality of life and enable patients to engage in emotionally and sexually satisfying relationships [2–5]. Various methods have been employed to construct a neovaginal canal [1, 6, 7]. Although no single technique is optimal, inversion vaginoplasty using penile-scrotal flaps is the most favored and widely practiced approach among surgeons [7]. However, there are instances where adequate penile-scrotal skin is unavailable due to anatomical constraints or patient expectations regarding vaginal depth. Moreover, it is increasingly common for younger individuals to undergo hormonal suppression in preparation for a gender transition [8].

Alternatives for vaginal reconstruction, such as full-thickness skin grafts [9], local flaps, musculocutaneous flaps [10–12], peritoneum [13–15], and various intestinal tissue segments, have been previously documented [16–19]. Intestinal vaginoplasty is a well-established method for addressing congenital or acquired vaginal absence [20]. In transgender patients, this technique is more frequently employed as a revision procedure following primary failure or complications, such as vaginal stenosis [21].

Recent pooled data analysis indicates that patients undergoing intestinal vaginoplasty are expected to face bowel complications related to the intestinal segment, barring which complication and mortality rates similar to those of penile inversion vaginoplasty, with several benefits [16]. Utilizing the intestinal segment ensures reliable achievement of sufficient depth of anesthesia. Intestinal grafts are less prone to shrinkage. Additionally, the mucosa resembles the vaginal mucosa in appearance and texture, with the added advantage of self-lubrication. Although elective bowel resection is often viewed as an unnecessary risk, recent data suggest fewer gastrointestinal complications in intestinal vaginoplasty than previously believed [9, 16]. Through this study, we offered Intestinal Vaginoplasty as a primary procedure for candidates who wanted sufficient vaginal depth and are motivated enough to withstand the complications and selected the procedure after being informed about both the options.

In this study, we present a retrospective series of 30 consecutive patients who underwent primary sigmoid vaginoplasty at our center between 2023 and 2025.

## Materials and methods

A prospective database was established to record information on patients who underwent primary sigmoid colon vaginoplasty at our center between 2023 and 2025. The collected data included baseline demographics, medical and surgical history, smoking status, complications, and postoperative vaginal depth. Vaginal depth was assessed using a dilator and reported in inches (in). Informed consent was obtained from all patients, including permission for the use of intraoperative photographs for publication purposes.

### Preoperative evaluation

We conducted a comprehensive physical examination, focusing particularly on any previous abdominal surgeries. In our practice, we advise that all individuals over 40 years of age undergo colonoscopy unless there are contraindications due to personal or family medical history. A high BMI was not considered a reason to avoid the procedure. On the day of or the day before the surgery, venous ultrasound/Doppler tests were performed on both the upper and lower limbs to check for deep venous thrombosis. In line with the WPATH guidelines, we instructed all patients to stop taking estrogen supplements 2-4 weeks before surgery. The day before the procedure, all patients underwent bowel preparation using Coloprep.

### Surgical procedure

At our facility, sigmoid vaginoplasty is performed in partnership with a surgeon who is an expert in extracting the pedicled sigmoid conduit for neovaginal construction. The operation utilized a concurrent abdominoperineal approach, with the patient positioned in the lithotomy position. To prevent infection, we routinely used perioperative Piperacillin-Tazobactam antibiotic to be administered, and an epidural may be administered to alleviate postoperative pain. Our mean operative time was 4 h 20 min, which is high compared to Penile Inversion Vaginoplasty which is 1 h 40 min. In case of a failed intestinal vaginoplasty, a revision or peritoneal and alternate sources were offered, although failure was not noted in our experience.

The procedure begins with a lower midline incision to open the abdominal cavity. The initial focus was on the sigmoid colon. Dissection was performed from the lateral to the medial side along the white line of Toldt, with careful identification and retraction of the ureter, while ensuring that the pelvic nerves were identified and preserved. The colon was mobilized up to the splenic flexure using both blunt and sharp dissection techniques, along with the LigaSure device.

**Figure 1:**
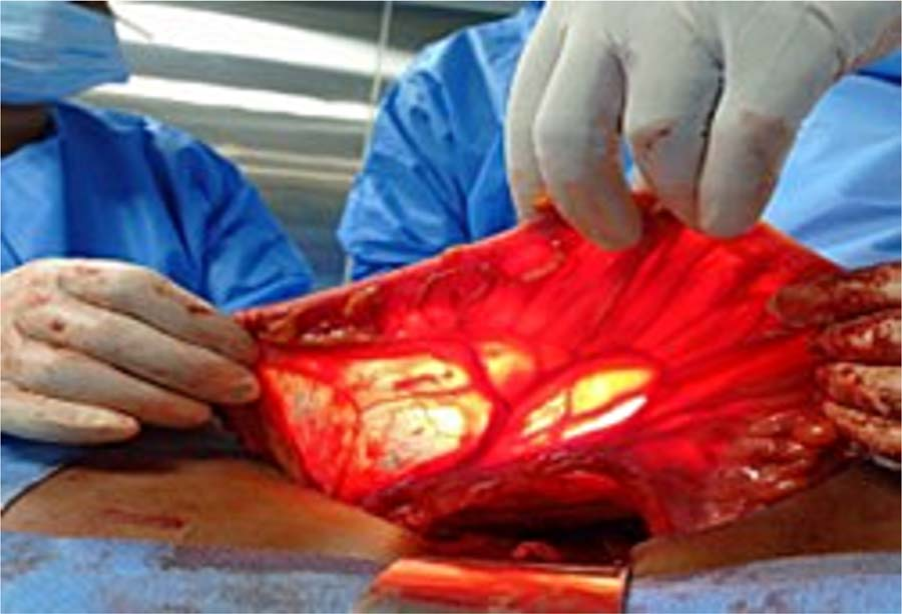
A Picture showing the assessment of sigmoid colon and vasculature.

**Figure 2:**
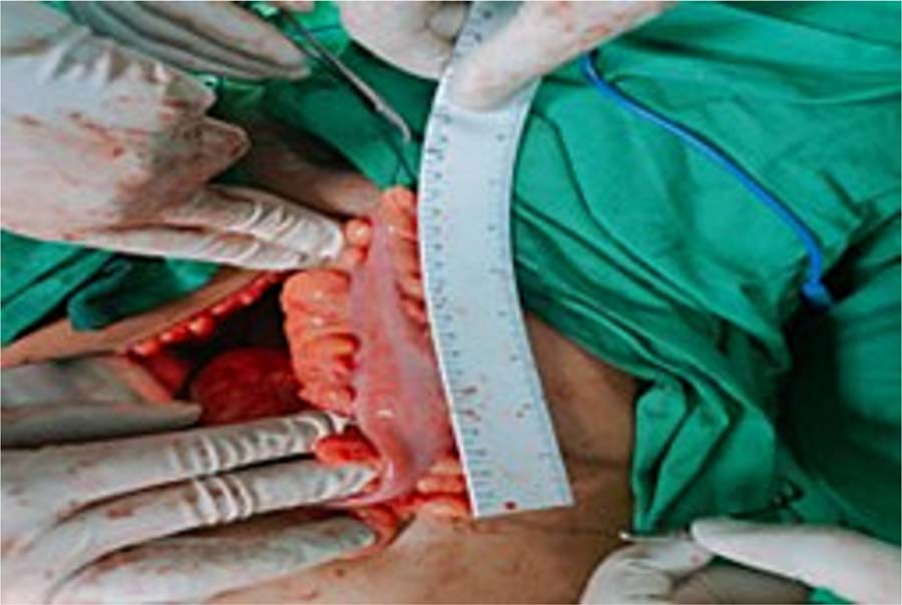
A Picture showing the measurement and selection of appropriate length.

**Figure 3:**
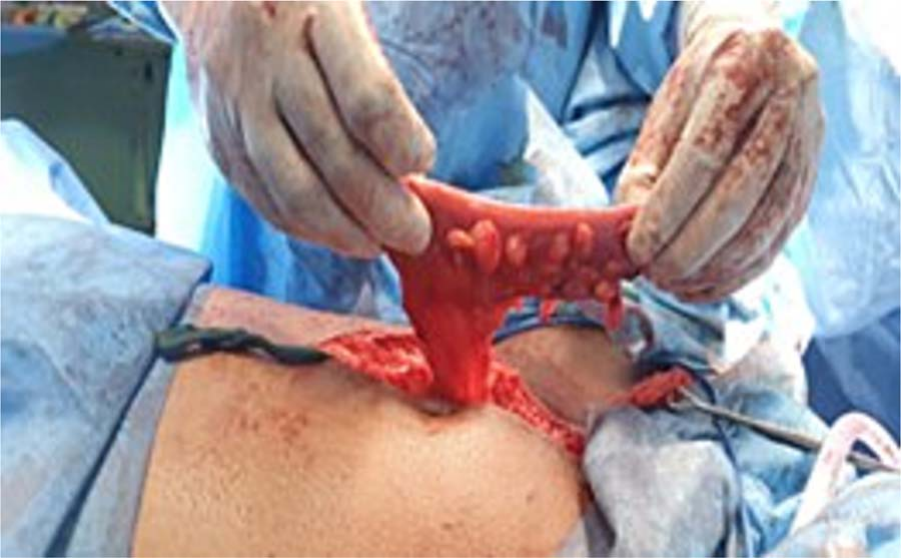
A Picture showing the length of the sigmoid colon resected.

**Figure 4:**
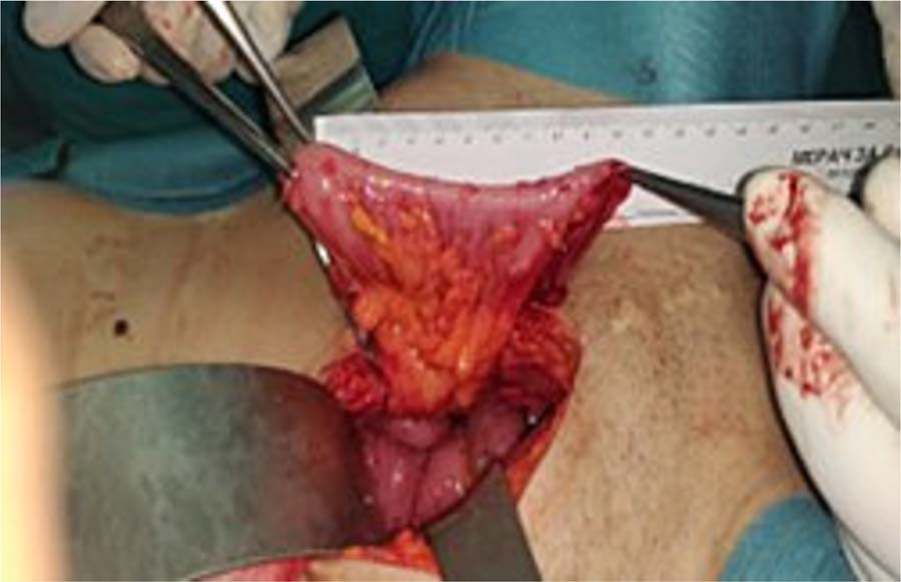
A Picture showing the final assessment of pedicle.

The sigmoid colon flap relies on vascular supply from the sigmoidal arteries, which branch off from either the inferior mesenteric artery (IMA) or the left colic artery (Figure 1). When the sigmoidal arteries stem directly from the IMA, the IMA is severed before the arteries. Conversely, if the sigmoidal arteries originate from the left colic artery, the IMA is cut before the left colic branch, and the left colic artery is cut beyond the sigmoidal arteries. The sigmoid loop, once mobilized, is measured and marked to a length of 15 to 17 cm for the flap, then transected using a linear stapler (Figure 2 and Figure 3). To confirm sufficient blood flow to the sigmoid conduit, indocyanine green can be injected intraoperatively, and an SPY imaging system can be employed. The proximal end was prepared for anastomosis by inserting the anvil of a circular stapler into the bowel and securing it with a purse-string suture. The pulsation of the pedicle was visually verified before it was returned to the abdominal cavity (Figure 4). The anastomosis is then completed using an end-to-end circular stapler, followed by a leak test using saline and air insufflation through the anus. Simultaneously, primary vaginoplasty and perineal dissection were initiated. An ellipsoid incision was made along the midline of the scrotal raphe, followed by bilateral orchidectomy. The penile skin was lifted from the neurovascular bundle and underlying corporal tissues. The neoclitoris is crafted from a section of the glans penis, carefully elevated from Buck’s fascia, ensuring that the dorsal penile nerves, deep dorsal artery, and veins are preserved.

A Foley catheter was inserted, and the corpora cavernosa were further exposed proximally to the corporal crura and individually tied off with sutures. Perineal dissection continued at the designated posterior fourchette, following an inverted U-shaped incision in the skin, while avoiding any damage to the rectum. Skin flaps were elevated along the inguinal crease to form the labia majora. Inside the abdomen, the peritoneum was cut using electrocautery to connect the abdominal and perineal dissections. The sigmoid conduit was passed through the neovaginal space in an antegrade manner, brought out for several centimeters, and secured with minimal tension at the level of the penile stump. Adequate mobilization of the sigmoid colon is generally achieved by releasing lateral attachments and performing a thorough mesenteric dissection after identifying the ureter and pelvic nerves, with the remaining sigmoid colon and rectum receiving blood supply from the middle and inferior rectal arteries, respectively. The penile skin is shortened to 1-2 inches to create the external genitalia. The penile stump is attached to the sigmoid conduit with absorbable Vicryl interrupted sutures, ensuring that the vascular supply prevents prolapse and resembles the appearance of a natal vaginal canal. The scrotal and inguinal skin are reshaped to form the labia majora, and the urethra is positioned cephalad to the introitus, spread open, and sutured in place. Clitoroplasty is performed using a triangular skin incision within the native mons pubis skin to create a clitoral hood, resulting in a final cosmetic appearance similar to penile inversion vaginoplasty.

### Postoperative care and follow-up

After surgery, patients typically stay in the hospital for 8 days postoperatively. Throughout this period, the neovagina was checked every 2 days with a speculum examination to ensure that the intestinal segment was clearly visible. Patients were encouraged to start walking after 48 h of bed rest. If an epidural is used for pain relief, it is usually stopped between the fourth and sixth postoperative day. The Foley catheter is kept in place for approximately 10 days and then removed during a follow-up office visit. Patients are advised to wait until after this appointment and the removal of the Foley catheter before beginning the dilation.

## Results

From 2023 to 2025, 30 patients underwent primary sigmoid colon vaginoplasty. The average age of the patients was 28 years (± 5.4 years), and their average BMI was 26.8 (± 5.6) as mentioned in Table 1. All patients were Indian and were undergoing estrogen therapy for gender transition. Overall, 80% (24 of 30) of patients had no intraoperative or postoperative complications. Three complications occurred: one patient experienced an anastomotic leak, and another had an intraoperative anal canal injury 3 cm from the anal verge, necessitating ileostomy and one patient had prolonged postoperative ileus. The reoperation rate was 3.4%, with one patient requiring surgery due to an anastomotic leak. Additionally, one patient (3.4%) developed vaginal stenosis at the neo introitus, attributed to defaulting the dilatation, which was successfully treated with dilation over the time. Detailed information on the complications and their management is outlined below. The minimum follow-up was 6 months with the first case being followed for 2 years.

**Table 1:** A table depicting the baseline, surgical and postoperative complication details of 30 patients.

Age in years (mean ± SD)	28 ± 5.4
BMI (mean ± SD)	26.8 ± 5.6
Intraoperative neovaginal depth	16 ± 2 cm
Neovaginal depth at follow up	14 ± 2 cm
Patients without complications	27/30 (90%)
Patients with complications	3/30 (10%)
Anastomotic leak-1	
Anal canal injury-1	
Prolonged ileus-1	
Introital stenosis, responded to dilatation	3/30 (10%)
Hospital stay (mean ± SD)	8 ± 2.1 days

### Complications and hospitalization

The typical duration of hospital stay was 8 ± 2.1 days. Due to geographical constraints rather than medical, 2 patients stayed at the Center for extra days. One patient encountered postoperative ileus, which was resolved through dietary adjustments. On the 10th day after surgery, another patient experienced widespread abdominal pain and an increased white blood cell count. A CT scan identified a 0.4 mm anastomotic leak, necessitating re-exploration and the creation of a diverting ileostomy. Furthermore, one patient developed a minor infection at the surgical site 2 weeks postoperatively, which was effectively treated with oral antibiotics.

Five weeks after the operation, another patient experienced mild introital stenosis. This condition was successfully managed with dilation under anesthesia, achieving a satisfactory vaginal circumference, and the patient continued with a regular dilation routine after the procedure. Two more patients had narrowing over the long term which was managed with dilatation. Importantly, there were no instances of diversion neo vaginitis, vaginal prolapse, sigmoid conduit necrosis, or rectovaginal fistula in the present series, which can be noted as a limitation owing to the sample size. Bowel Complications of anastomotic leak and ileus also can be considered a limitation due to small sample size.

### Outcomes

The follow-up period ranged from 6 months to 18 months postoperatively, with evaluations carried out either via phone calls or in-person clinic visits, depending on the location of the patients. At the final follow-up, the average depth of the neovagina was 5.5 ± 0.8 inches (14 ± 2.0 cm). All patients indicated that they could participate in vaginal intercourse and were satisfied with both the sensation and depth of their neovagina. The depth achieved was sufficient for penetrative intercourses. Furthermore, none of the patients reported unpleasant odors or excessive secretions from their neovaginas.

## Discussion

Sigmoid vaginoplasty is a reliable surgical option with minimal complications that provides satisfactory vaginal depth in transgender women. In our practice, we have detailed conversations with patients about their expectations regarding penetrative intercourse, anatomical factors, and desired results before proceeding with sigmoid vaginoplasty. We customize vaginal depth to suit each individual’s needs rather than adhering to a standard measurement. The patients requested a certain depth according to their needs for sexual outcomes and self-lubrication and the length was planned accordingly. The procedure involves isolating a section of the sigmoid colon while preserving the blood supply from the sigmoid arteries. This section is usually placed in an isoperistaltic orientation and attached to the penile-scrotal structures of the neovagina using interrupted sutures [22].

Alternatives such as the ileum and cecum have also been utilized; however, positioning the cecum without tension is difficult because of its limited mesentery and anatomical position. Compared to the ileum, the sigmoid colon generates fewer secretions and more closely resembles the vaginal circumference without requiring additional surgical alterations [23].

The benefits of using the sigmoid colon over full-thickness skin grafts include consistently achieving sufficient vaginal depth, a natural-looking mucosa that produces its own secretions, and lower rates of widespread vaginal stenosis. It is crucial to inform patients that the colonic segments still require postoperative dilation to prevent stenosis at the neovaginal introitus. The recommended dilation period is 6-12 months after surgery to avoid introital stenosis. Over time, patients typically require less frequent dilations. This procedure can be done in Robotic approach when and where available, further adding to the reduced morbidity of Laparotomy incision and dissection. In our experience, the patients had a low average BMI compared to pooled data, which can avoid the difficulty of passing sigmoid through the pelvic inlet due to high peri colonic fat.

However, drawbacks include the need for abdominal surgery and bowel anastomosis. Alternatives, such as omental and peritoneal flaps, maintain bowel continuity and may reduce operative time and hospital stay. However, these flaps require extensive surgical manipulation to create a neovaginal canal from the source compared to colon, and healing can be unpredictable. Peer-reviewed studies on peritoneal grafts in transgender women are limited [24, 25].

Another drawback noted is the extra days of admission required for Preoperative insurance authorization and legal processing as patients came from long distances and prolonged postoperative stay till, they are comfortable to travel long, which added to the mean hospital stay.

Research has shown that transgender women who undergo sigmoid vaginoplasty experience considerable satisfaction in terms of both sexual function and appearance after surgery. Our prospective study outlines the surgical results of 10 patients who underwent primary sigmoid colon vaginoplasty at our institution. The study was limited by the small sample size and a follow-up period of 6 months. Many patients traveled long distances for surgery, making extended follow-up challenging. Despite these obstacles, our technique consistently results in sexually functional neovaginal canals with satisfactory depth and size [20].

The average vaginal depth post-surgery in our study was 6 ± 0.8 inches (15 ± 2.0 cm), which is consistent with the 11.5-13.0 cm range reported in other studies. All sexually active participants in our study reported sufficient depth of sexual satisfaction. We observed 3 cases of introital stenosis (10%) that was successfully treated with dilation. This incidence is similar to the 8.6% stenosis rate in pooled data and 14.6% in a recent study by Bouman et al. Regular dilation is generally effective in treating stenosis, which usually occurs within the first year after the surgery. Our major complication rate was 10% excluding stenosis which settled with dilatation, compared to 6.4% in the pooled data [26–31]. None of the patients reported unpleasant odor over 6 months, although pooled data says otherwise, this was a finding we noted in our study.

We encountered minimal intraoperative or postop [32] erative abdominal complications, likely due to the simultaneous intra-abdominal and perineal procedures that enhanced the visualization and safe retraction of vital structures, thereby reducing the risk of bowel injury. In line with the WPATH SOC criteria, we required 12 continuous months of hormone therapy before performing genital surgery in male-to-female transgender patients [33, 34]. However, due to short follow-up, we didn’t have long term complications related to dilation, diversion colitis, cancer [35].

## Conclusions

Sigmoid vaginoplasty is a dependable method that closely resembles the natural vaginal lining and offers a satisfactory tactile experience, ensuring sexual function and patient satisfaction. Achieving optimal results necessitates a collaborative effort among a proficient surgeon, transgender healthcare team, urologist, and plastic surgeon.
